# Davanat-Mimetic Galactomannan and Its Sulfated Derivative: Structure and Antitumor Effects against Melanoma

**DOI:** 10.1021/acs.biomac.5c00290

**Published:** 2025-08-21

**Authors:** Odair Braz Júnior, Aline Miranda Cristal, Daniel de Lima Bellan, Gustavo Rodrigues Rossi, Stellee Marcela Petris Biscaia, Camila Laís Gonçalves Ribeiro, Jacqueline Gonçalves dos Santos, Anderson Fraga da Cruz, João Luiz Aldinucci Buzzo, Helyn Barddal, Thales R. Cipriani, Marcelo Dias-Baruffi, Edvaldo da Silva Trindade, Fernanda Fogagnoli Simas, Carolina Camargo de Oliveira

**Affiliations:** † Cell Biology Department, 28122 Universidade Federal do Paraná (UFPR), Curitiba, 81.531-980, Brazil; ‡ Biochemistry Department, Universidade Federal do Paraná (UFPR), Curitiba, 81.531-980, Brazil; § School of Pharmaceutical Sciences of Ribeirão Preto, 28133 Universidade de São Paulo (USP), Ribeirão Preto, 14040-903, Brazil

## Abstract

Melanoma is the most aggressive skin cancer, with a high metastatic potential and limited treatment options in advanced stages. Polysaccharides are promising antitumor agents, and therefore, this study investigated a galactomannan from guar gum hydrolysis (GGH) and its sulfated derivative (GGHS) for their antimelanoma and immunostimulatory effects. GGH shares structural similarity with DAVANAT, a galectin-1 ligand with anticolorectal cancer activity, while GGHS has anticoagulant properties, like heparin used in cancer patients. *In vitro*, 100 μg/mL GGH or GGHS inhibited melanoma cell invasion, increased adhesion, and reduced colony size, while GGHS also reduced proliferation. Both compounds bind galectin-3 and -1, but only GGH suppressed tumor progression in mice. Both treatments stimulated macrophage proinflammatory responses, including reactive oxygen species production and cytokine secretion. Although *in vitro* lymphocyte proliferation was not observed, CD3+ cells increased in the metastatic lungs. These results suggest GGH and GGHS as immunostimulatory agents, with GGH as potential melanoma adjuvant therapy.

## Graphical abstract

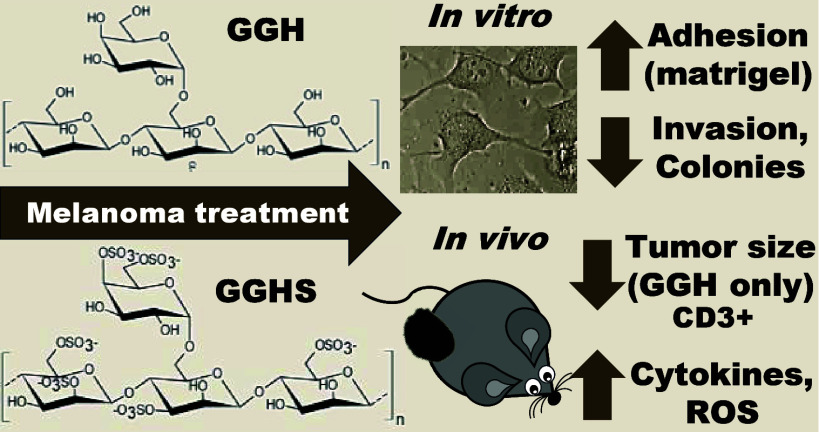

## 1 Introduction

Melanoma is the most aggressive type of skin cancer due to its high metastatic potential. The global incidence is expected to rise with increasing UV exposure and environmental changes. In 2022, 331647 new cases and 58645 deaths were reported worldwide, and projections suggest up to 510000 cases and 96000 deaths by 2040.^1^

The skin and lungs are the most frequent metastatic sites. During the metastatic process, melanoma cells invade and extravasate from the circulation. These processes involve upregulation of adhesion molecules on melanoma cells surface, metalloproteinases alteration, and vascular recognition mechanism.^2^ Galectins are glycan-binding proteins which are overexpressed in melanoma cells and facilitate heterotypic interactions with lung endothelial cells via glycosylated surface molecules accounting for selective metastasis^3^ and a worse prognosis.^4^ In addition, they have been implicated in tumor defense enhancement, reducing CD4+ and CD8+ T-cell infiltration and inducing apoptosis of these cells.^5^ Melanoma progression is closely related to its microenvironment modulation, especially regarding the immune system response.^6^ A range of cytokines and chemokines regulate immune cells recruitment and function.^7^ MCP-1 is the primary chemokine for recruiting lymphocytes and macrophages to the tumor, and its presence can lead to both tumor reduction and/or progression.^8^ INF-γ and TNF-α play a crucial role in the elimination of melanoma cells by inducing an antitumor response.^7^ On the other hand, molecules such TGF-β, IL-6, IL-10, and IL-12, play a protumor role in melanoma by triggering an immunosuppressive stimulus in immune cells.^9–11^

Current treatment for advanced melanoma was greatly improved by target and immunotherapy.^12^ However, intrinsic or acquired resistance to therapy still impairs advances in increasing overall patient survival rates.^13,14^ Additionally, the adverse effects limit therapies continuation.^15^ Therefore, the development of less toxic interventions remains critical. Targeting cellular and molecular components of the tumor microenvironment represents a promising area to mitigate melanoma progression and enhance therapeutic outcomes.

Polysaccharides from different sources were demonstrated by our group^16–19^ and by other authors^20–24^ to possess antimelanoma activity. They can reduce melanoma tumor volume and metastasis foci by changing melanoma cells phenotype and tune the immune system against tumor development and progression.^25,26^ In this context, galactomannans are a group of polysaccharides that consist of a mannose backbone with galactose side chains that present antimelanoma effects both *in vitro* and *in vivo*. PSP001 (from *Punica granatum* with a molecular weight of 110 kDa) was shown to reduce malignant characteristics in murine (B16–F10) and human (A375) melanoma cells, inhibiting invasion, migration, and colony formation, modulating metalloproteinases expression, and reducing the development of melanoma lung metastasis.^27,28^ It is known that structural modifications in galactomannans can add new properties to these molecules as well as can abrogate their biological activities.^29^ Guar gum, another galactomannan-type of polysaccharide extracted from *Cyamopsis tetragonoloba* seeds and well-known for its industrial use as food additives, have shown biological activities,^30^ especially when chemically modified. Guar gum glycosylated and sulfated increased proliferation and phagocytic capacity of macrophages (RAW 264.7), as well as reduced proliferation of hepatocarcinoma (HepG2) and breast cancer (MCF-7) cells.^31^ DAVANAT (a partially hydrolyzed guar gum with a molecular weight of 42–60 kDa) has been shown to inhibit tumor growth and increase survival in *in vivo* models of colorectal cancer. Studies suggest that its effects are primarily mediated through the immune system by binding to galectin-1 and -3.^32,33^

Sulfated polysaccharides are interesting molecules regarding melanoma therapy since they could both interact directly with tumor cells, inducing apoptosis, cell cycle arrest, decreasing invasion capacity and with extracellular matrix interacting with heparanases and metalloproteinases.^18,34–38^ Additionally, sulfated polysaccharides can exhibit anticoagulant effects, which is also interesting in cancer therapy. Cancer patients have a high probability of developing thromboembolism due to the ability of tumor cells to activate the blood coagulation cascade.^39^ These patients are usually treated with heparin, which has been shown to increase their survival time. In addition to its anticoagulant action, low molecular weight heparin can inhibit platelet aggregation on tumor cells surface in the bloodstream promoting an increase in immunosurveillance.^40^ Some studies have also reported direct effects of heparins, desulfated heparins, and low molecular weight heparins on tumor cells.^41–43^

Considering all the above, the effects on melanoma of a partially hydrolyzed derivative of guar gum, structurally similar to DAVANAT (hGG, here denominated GGH) and its sulfated derivative (hGGSL, here denominated GGHS) were evaluated. These molecules were previously synthesized, characterized, and chemically sulfated by Barddal et al. 2020.^44^ The sulfated derivative was tested for its anticoagulant and antithrombotic properties, showing positive results, albeit with a lower potency than heparin. This characteristic is particularly interesting for its potential application as an antitumor agent, as the strong anticoagulant effect of heparin can lead to severe hemorrhagic complications.^45^ Therefore, the current study aimed to evaluate their *in vitro* and *in vivo* effects on melanoma cells, as well as their impact on tumor microenvironment, with a particular focus on immune system cells such as lymphocytes and macrophages was also evaluated.

## 2 Materials and Methods

### 2.1 Polysaccharides: Synthesis, Characterization, and Preparation for Biological Assays

The GGH polysaccharide was produced according to Bardall et al.^44^ Guar gum (5 g) was partially hydrolyzed with 0.5 M TFA (600 mL) at 100 °C for 2 h. After neutralization with 10% NaOH (w/v) the solution was exhaustively dialyzed against distilled water (6–8 kDa cutoff membrane). The dialysis was finalized when no more carbohydrates were detected on dialysis water and when dialysis water showed conductivity similar to ultrapure water (0.06 μ/cm Siemens). The solution was freeze-dried to yield partially hydrolyzed guar gum (GGH; 16.4% yield; 0.822 g).

The GGH chemical sulfation was carried out according to Larm et al.^46^ GGH (150 mg) was dissolved in *N*,*N*-dimethylformamide (10 mL) and reacted with pyridine-sulfate complex (18 mol for each mole of free hydroxyl group). After 6 h of reaction at 25 °C, the reaction was stopped with 10% aq. NaHCO_3_ until pH 7.0, dialyzed against ultrapure water (6–8 kDa cutoff membrane) until dialysis water have shown conductivity like ultrapure water (0.06 μSiemens/cm) and the retained polysaccharide was freeze-dried yielding GGHS (261 mg).

Polysaccharides *M*_w_ was determined by high performance size exclusion chromatography (HPSEC). The analyses were performed using four Ultrahydrogel columns in series (7 × 10^6^, 4 × 10^5^, 8 × 10^4^, and 5 × 10^3^ Da exclusion limits) connected to a refractive index detector. The eluent was 0.1 M aq. NaNO_2_. Polysaccharides (1 mg/mL) were filtered through a 0.22 μm (Millipore) nitrocellulose membrane before injection (100 μL loop). The relative *M*_w_ was estimated to use a dextran standard curve (standard dextrans with *M*_w_ of 5, 9.4, 17.2, 40.2, and 72.2 × 10^3^ Da) (Supporting Information - Figure S1).

The degree of substitution (DS) of GGHS by sulfate groups was quantified by the BaCl_2_-gelatin turbidimetric method, using Na_2_SO_4_ as standard,^47^ after acid hydrolysis of the GGHS (1 M HCl at 110 °C for 5 h). The degree of substitution (DS) was obtained according to the equation described by Whistler et al.^48^

The 2D-NMR HSQC-DEPT correlation maps were obtained with a Bruker 600 MHz AVANCEIII NMR spectrometer with a 5 mm inverse gradient probe head (QXI). Analyses were carried out at 50 °C on samples (10 mg) dissolved in D_2_O. Chemical shifts were expressed in δ relative to acetone at δ 2.21 (^1^H) and 30.2 (^13^C).

For the *in vitro* assays, stock solutions of 1000 μg/mL in DMEM or RPMI-1640 culture media were prepared by magnetic stirring and sterilized by filtration in 0.22 μm nitrocellulose membrane (Millipore), and lower concentrations were obtained by serial dilutions. For the *in vivo* assays, the stock solutions were prepared at 1 mg/mL in sterile PBS, sterilized as described above, and administered to achieve a daily dose of 5 mg/kg.

### 2.2 *In Vitro* Assays Using Cell Lines

Murine melanoma B16–F10 cells (ATCC BCRJ, 0046) and BALBc/3T3 clone A31 fibroblasts (ATCC CCL-163) were maintained in Dulbecco’s Modified Eagle Medium (DMEM; Gibco), supplemented with 10% fetal bovine serum (FBS; Gibco), 1.5 g/L sodium bicarbonate, and the antibiotics, 1 U/mL penicillin and 1 μg/mL streptomycin (Life Technologies), at 37 °C in a humid atmosphere with 5% CO^2^. Subculturing was performed when the cells reached a maximum confluence of 80% of the culture area using trypsin-EDTA (Gibco).

Jurkat T lymphocyte clone E6 (ATCC BCRJ, 0125) were cultured in 75 cm^2^ culture flasks (Sarstedt) with RPMI-1640 medium (Gibco), supplemented with 10% inactivated fetal bovine serum (Gibco) and 0.25 μg/mL of penicillin-streptomycin antibiotics (Gibco) in 0.85% saline solution. They were maintained in an incubator with 5% CO_2_ at 37 °C in a humid atmosphere for no more than five passages after thawing.

#### 2.2.1 Cytotoxicity and Proliferation Assays

To evaluate whether the polysaccharides altered cell viability, the neutral red assay was performed.^49^ Four hundred melanoma cells and two thousand fibroblasts cells were cultured in 96-well plates (Sarstedt) during 24 h. Cells were treated with GGH and GGHS at concentrations of 0.0001, 0.001, 0.01, 0.1, 1, 10, and 100 μg/mL for 72 h. After treatment, culture medium was removed, and the cells were incubated for 2 h with 0.04 mg/mL of neutral red (Sigma-Aldrich). The medium was then removed, and an extraction solution containing ethanol, water, and glacial acetic acid (50:48:2 v/v) was added. Absorbance was measured at 540 nm using a microplate reader (BioTek Instruments).

To measure cell density, the crystal violet staining method was used.^50^ After reading the neutral red absorbance, supernatants were removed, and the fixed cells were washed with distilled water and incubated with 0.25 mg/mL crystal violet solution for 20 min. The plate was washed, and the dye was eluted with a 33% acetic acid solution. Absorbance was measured at 570 nm (BioTek Instruments).

#### 2.2.2 Cell Morphological Analyses by Scanning Electron and Confocal Microscopies

To evaluate whether the treatments altered cell morphology, melanoma cells were cultured in 24-well plates (Sarstedt) on circular glass coverslips (Knittel Glass), and on Matrigel-coated coverslips. After 72 h of treatment with 100 μg/mL GGH or GGHS, cells were washed, fixed with glutaraldehyde, and postfixed with osmium tetroxide. After fixation, cells were dehydrated in ethanol, CO_2_ critical point dehydrated (CPD 010 Critical Point Dryer Balzers), gold-coated (SCD 030 Balzers), and analyzed using a scanning electron microscope (JEOL JSM 6360), with images captured at magnifications of 200 and 800 times.

For confocal microscopy, melanoma cells were fixed and stained with DAPI and phalloidin-Alexa Fluor 488. The cells were washed once with PBS at 37 °C and fixed with 2% PFA in PBS for 30 min. The cells were stained with 50 μL per coverslip of a PBS solution containing 0.01% saponin, 1 μg/mL DAPI (Thermo Fischer), and ActinGreen (one drop per 2 mL; ActinGreen 488 ReadyProbes Reagent, Thermo Fischer) for 30 min at room temperature. After staining, the coverslips were mounted with 10 μL of Flourmount G (E.M.S), images were obtained using a laser scanning confocal microscope system (A1R MP+ Nikon) and analyzed with ImageJ Fiji software.

#### 2.2.3 Cell Death and Cell Cycle Analyses

Cell death was analyzed by flow cytometry using an apoptosis detection kit (BD Biosciences) according to manufacturer recommendations. Twelve thousand melanoma cells were plated in 6-well plates (Sarstedt) and treated with 100 μg/mL GGH or GGHS for 72 h. The supernatants were then collected and reserved. Adherent cells were detached with 2 mM EDTA in PBS for 5 min and mixed with their respective reserved supernatants. Cells were centrifuged and, after being counted, 500000 cells were resuspended in 100 μL of Annexin V binding buffer diluted 1:10 in PBS, along with 2.5 μL of Annexin V-FITC and 5 μL of PI (BD Pharmingen, 559763). They were incubated for 15 min on ice in the dark. After incubation, 400 μL of binding buffer was added, and samples were analyzed by flow cytometry using a FACSCalibur (Becton, Dickinson and Company, BD), acquiring 20000 events per group. As a positive control for apoptosis, cells were incubated with 10 μM camptothecin (Sigma-Aldrich) during the last 18 h of culture, and for the positive control of necrosis, cells were incubated with 0.01% saponin in PBS for 30 min before processing.

Melanoma cells were plated in 6-well plates (Sarstedt; 18000 cells per well) and incubated for 24 h in DMEM (Gibco) deprived of FBS to synchronize the cells cycle. Next, cells were exposed to polysaccharides at 100 μg/mL for 72 h in DMEM (Gibco) with 10% FBS (Gibco). Cells were detached with 2 mM EDTA (Gibco), washed twice with PBS, resuspended in 70% ethanol, and maintained in −20 °C for 24 h. Before flow cytometry analysis, cells were washed twice with PBS and incubated with a PI/RNase (550825, BD Pharmingen) staining kit for 20 min. A total of 20000 events per group were analyzed on a FACSVerse flow cytometer (Becton, Dickinson and Company, BD), and the data were processed using Flowing software.

#### 2.2.4 Invasion Assay

B16–F10 cells were pretreated in 6-well plates (Sarstedt; 12000 cells per well) with 100 μg/mL GGH or GGHS for 72 h. Cells were then detached using 2 mM EDTA (Gibco) in PBS for 5 min and resuspended in DMEM (Gibco) culture medium without FBS. Subsequently, 80000 cells were seeded into inserts with 8 μm pore polycarbonate membranes (Thincert Cell Culture Insert For 24 Well Plates – 662638 - Greiner), coated with 35 μL of Matrigel (2.5 mg/mL), in 24-well plates. Outside the inset, in the wells, DMEM culture medium containing 10% FBS was added, acting as a chemoattractant to induce cell invasion into the inset. The plates were incubated for 72 h at 37 °C and 5% CO_2_. After this period, the insets were fixed with 2% v/v paraformaldehyde in PBS for 30 min. The cells were stained with 50 μL of a PBS solution containing 0.01% saponin, 1 μg/mL DAPI (Thermo Fischer), and ActinGreen (one drop per 2 mL) (ActinGreen 488 ReadyProbes Reagent, Thermo Fischer), for 30 min at room temperature. Subsequently, noninvading cells were removed using a cotton swab (upper insert side), the insets were cut with a scalpel, and the slides were mounted with Fluoromount G, with the bottom side insert facing up (containing the invading cells).

Image acquisition was performed using an Axio Imager Z2 fluorescence microscope (Carl Zeiss), equipped with the automated capture software Metafer 4/VSlide (Metasystems), using a 20x objective lens. The analysis consisted of counting the number of cells (using nuclear staining), and this number was divided by the inset area using the Fiji ImageJ software.

#### 2.2.5 Cell Adhesion Assay

For the adhesion assay, the wells of a 96-well plate (Sarstedt) were sensitized with 50 μL of 20 μg/mL Matrigel in PBS overnight at 4 °C. As a control, some wells remained uncoated.

Melanoma cells were pretreated in 6-well plates (Sarstedt) with 100 μg/mL GGH or GGHS for 72 h. They were then detached using 2% EDTA in PBS for 5 min and resuspended in DMEM with 10% FBS. After counting, 24000 cells were plated in each well and incubated for 2 h and 30 min at 37 °C with 5% CO_2_. The wells were washed once with PBS at 37 °C, and the adhered cells processed for crystal violet staining, as described in section 2.2.1.

#### 2.2.6 Clonogenic Assay

To verify melanoma cells clonogenic capacity, 400 cells were plated in 6-well plates (Sarstedt), and after 24 h, they were exposed to 100 μg/mL GGH or GGHS for 96 h. After that, cells were washed with PBS, fixed with a solution of ethanol, distilled water, and glacial acetic acid (50:48:2 v/v), washed again with PBS, and stained with 0.25 mg/mL crystal violet for 30 min.^51^ After, the wells were washed with distilled water, and the size and number of colonies formed were evaluated through photographs, which were analyzed using Fiji ImageJ software.

#### 2.2.7 Lymphocytes Cell Proliferation Assay

To evaluate GGH and GGHS effects on lymphocytes proliferation, 2.6 × 10^6^ Jurkat cells were resuspended in 5 mL of PBS (Gibco) with 5% FBS and incubated with 50 μL of carboxyfluorescein succinimidyl ester (CFSE; BD - Becton, Dickinson and Company, EUA) for 10 min at 37 °C. After adding RPMI-1640 (Gibco) medium with 5% FBS (Gibco) and incubating for 5 min, the cells were centrifuged, resuspended, and plated at 1 × 10^5^ cells/mL in 24-well plates (Sarstedt). Cells were then treated with 100 μg/mL GGH or GGHS, or 5 μM mitomycin (Sigma-Aldrich), or left untreated, for 96 h. Afterward, the cells were fixed with 2% paraformaldehyde and transferred to flow cytometry tubes for analysis using the BD FACSMelody Cell Sorter (Becton, Dickinson and Company, BD) and FlowJo software.

### 2.3 Galectin-1 and -3 Binding Assay

Human Gal-1 and Gal-3 were produced in *Escherichia coli* BL21/DE3 transformed with plasmid pET11a, encoding the human Gal-1 and Gal-3 gene, and thus produced and purified as described in Biscaia et al., 2022.^52^ The binding between polysaccharides and Gal-1 and Gal-3 was analyzed on a Quartz Crystal Microbalance (QCM-D) E4 instrument (Biolin Scientific AB). The entire process of binding the Galectin to the sensors, as well as the protocol used, was based on Biscaia et al., 2022.^52^

### 2.4 *In Vivo* and *Ex Vivo* Biological Assays

Animals used were male C57BL/6 mice, aged between 8 and 12 weeks, and maintained in the animal facility of the Biological Sciences Department at UFPR. The experiments were conducted after project approval by the Ethics Committee for Animal Use of the Biological Sciences Department from UFPR (CEUA-BIO UFPR No. 1389).

#### 2.4.1 Isolation and Polarization of Bone Marrow Derived Macrophages from Healthy Mice

Bone marrow from healthy C57BL/6 mice (*n* = 5) was collected under sterile conditions. The cells were processed according to Heap et al., 2021.^53^ Briefly, the pellet was incubated on ice for 5 min in RBC lysis buffer, washed by centrifugation, and pellet was resuspended in 2 mL of differentiation medium (DMEM with stable glutamine (Gibco) + 20% FBS (Sigma-Aldrich) + 20% L929 conditioned media + 20 μg/mL gentamycin (Gibco) and centrifuged (36000 g). The L929 fibroblast cell line used to prepare the conditioned medium is a well-established source of macrophage colony-stimulating factor (M-CSF), which plays a crucial role in the differentiation and survival of bone marrow-derived macrophages (BMDMs) *in vitro*.^53–55^ A total of 6 × 10^6^ cells were plated in 6-well plates with 8 mL of differentiation medium and incubated for 7 days at 37 °C with 5% CO_2_, the culture medium being changed on day 5. On day 7, cells (M0) were detached and counted with Trypan Blue, and 2 × 10^6^ cells were plated on a 6 well plate (Sarstedt). M0 cells were kept in DMEM with 20% fetal bovine serum and 20 μg/mL gentamycin. After 48 h, M0 cells were treated with 100 μg/mL GGH or GGHS for 72 h, and immunophenotyping assay was performed. M1 polarization was induced with 20 ng/mL IFNγ (Invitrogen, EUA) and 100 ng/mL LPS (Sigma-Aldrich) as a control.^55^

#### 2.4.2 Solid Tumor and Experimental Metastasis Mouse Models

B16–F10 melanoma cells were detached from culture flasks at 70–80% confluence using 1% trypsin (Sigma-Aldrich) in PBS (Gibco), resuspended in DMEM (Gibco), centrifuged, and washed twice in PBS. Viability was determined using Trypan Blue dye (Sigma-Aldrich). A total of 100 μL containing 5 × 10^5^ cells were injected subcutaneously on the dorsal flank of mice to form a solid tumor, as well as into the tail vein for experimental metastasis model.

Five days of postinoculation, mice were daily treated with 5 mg/kg GGH or GGHS, or with PBS (control group) through intraperitoneal injections for 10 days. Tumor measurements were taken with calipers, and tumor volume was calculated using an elliptical formula “V = dxdxDx0.52” (“d” = smaller tumor dimension, “D” = bigger dimension). At the end of the experiment, mice were weighed and anesthetized for peritoneal lavage (to isolate macrophages) and blood collection for biochemical tests, then euthanized by cervical dislocation. Organs and tumors were collected. For histological analyses, fixed tumors were divided in half and dehydrated in increasing concentrations of ethanol, cleared in xylene and embedded in paraffin. Subsequently, 5 μm histological sections were made in a microtome and deposited on slides to carry out staining techniques hematoxylin and eosin (H&E).^60^ Finally, the slides were mounted with an Entellan (Merck). The material images were acquired using a Histological Slide Scanner (model Vslider, CarlZeiss/Metasystems). The other half of the lung and tumors were preserved in DMEM with fetal bovine serum at 7 °C overnight for further immunophenotyping and cytokine quantification. The metastatic area in the lungs was quantified by using image analysis software (ImageJ).

##### 2.4.2.1 Tumor, Lungs, and Peritoneal Macrophages from Melanoma-Bearing Animal Cells: Obtaining and Processing

After being collected, the lungs and tumors were stored overnight in DMEM (Gibco) with 10% heat-inactivated FBS at 7 °C in 24-well plates. On the following day, they were cut into smaller pieces and exposed to a digestion solution containing 1.5 mg/mL *Clostridium histolyticum* collagenase (Sigma-Aldrich), 5% heat-inactivated FBS, and 10 mM HEPES (Sigma-Aldrich) for 45 min at 37 °C. After digestion, the samples were homogenized, centrifuged to remove the digestion solution, and passed through a 70 μm cell strainer. They were then treated with red blood cell lysis buffer on ice, centrifuged again, and resuspended in PBS for immunophenotyping. For cytokine quantification in lung and tumor, both were directly passed through a 70 μm cell strainer (Corning). Samples were aliquoted into 2 mL microtubes and centrifuged, and the supernatant was collected for subsequent cytokine quantification processing.

Peritoneal macrophages from melanoma-bearing animals were collected from C57BL/6 anesthetized mice. After the peritoneal area was exposed, 10 mL of PBS at 4 °C was injected into the peritoneal cavity, and after massaging the area, the contents were carefully withdrawn and kept in a 15 mL centrifuge tube stored on ice. Samples were centrifuged and resuspended in 5 mL of RPMI 1640 medium with 10% inactivated fetal bovine serum and 0.25 μg/mL (Sigma-Aldrich) of penicillin-streptomycin antibiotics (Gibco). A differential count was performed in a Neubauer chamber using Trypan Blue dye (Sigma-Aldrich), 2 × 10^6^ cells were plated per well in 6-well plates and 1 × 10^5^ cells per well in 96-well plates (Sarstedt). Plates were maintained in a humidified incubator with 5% CO_2_ at 37 °C for 24 h. After this period, the cells were exposed to 100 μg/mL GGH or GGHS for 72 h, and nontreated cells were maintained as control.

#### 2.4.3 Immunophenotyping

Lymphocyte profile analyses in lung and tumor samples were performed by using the antibodies CD3 (PerCP Cy 5.5, Invitrogen) to identify the general lymphocyte population; CD4 (PE - Invitrogen) to identify regulatory T lymphocytes; CD8 (PE - Invitrogen) to identify cytotoxic T lymphocytes; and CD25 (FITC - Invitrogen) to identify their activation.

Bone marrow-derived macrophage samples were processed for immunostaining. Macrophages were identified by the presence of F4/80 (APC eFluor 780 - Invitrogen) marker. To identify M1 macrophage phenotype, the antibodies CD80 (PerCP efluor 710 - Invitrogen) and CD86 (APC - Invitrogen) were used (Supporting Information, Figure S2). Data were acquired on a FACSMelody flow cytometer (BD Biosciences) using the appropriate laser channel, with 50000 events for each lung and tumor sample and 8000 events for bone marrow macrophages. Data analysis was performed by using FlowJo software.

#### 2.4.4 Cytokine Analyses

To quantify the cytokines present in the lung, tumor, and supernatant from macrophages obtained by peritoneal lavage of melanoma-bearing mice, the BD Cytometric Bead Array (CBA) Mouse Inflammation kit was used for detection of the following cytokines: Interleukin-6 (IL-6), Interleukin-10 (IL-10), Monocyte Chemoattractant Protein-1 (MCP-1), Interferon-γ (IFN-γ), Tumor Necrosis Factor (TNF), and Interleukin-12p70 (IL-12p70). The data were obtained using the FACSCalibur flow cytometer (BD Bioscience), employing the CellQuest software (BD Biosciences) with 2100 events recorded for each sample. Data analysis was performed using FCAP Array software (BD Biosciences).

#### 2.4.5 Reactive Oxygen Species (ROS) Assay

To quantify reactive oxygen species in peritoneal macrophages from melanoma-bearing mice, the fluorescent probe DCFH-DA (Sigma-Aldrich) was used. After plating and *ex vivo* exposure of macrophages to 100 μg/mL GGH or GGHS for 72 h, the culture medium was removed from the 6-well plates and washed with Hanks balanced salt solution (HBSS). Cells were exposed to 1 mL of 10 μM DCFH-DA in HBSS for 30 min at 37 °C and 5% CO_2_. After incubation cells were detached from the plates using a cell scraper and transferred to 12 mm × 75 mm flow cytometry tubes. The data were obtained using the FACSMelody flow cytometer (BD Bioscience) on the appropriate laser channel, with 8000 events recorded for each sample. Data analysis was performed using FlowJo software.

### 2.5 Statistical Analysis

At least 4 independent *in vitro* experiments were performed with technical replicates. Three independent *in vivo* experiments were performed, with a final *n* of Control-PBS, *n* = 15; 5 mg/kg GGH, *n* = 11; 5 mg/kg GGHS, *n* = 16. Significant differences between experimental and control groups were determined by parametric statistical tests, which are described in the respective figure legend. Statistical analyses were performed using *GraphPad Prism* 6. *P*-values < 0.05 were considered statistically significant.

## 3 Results and Discussion

### 3.1 Synthesis and Characterization of Polysaccharides GGH and GGHS

GGH and GGHS were produced according to Barddal et al.^44^ and they were obtained with similar structure, molar mass, and sulfation profile similar to those previously produced. Both GGH and GGHS showed a monomodal homogeneous profile by HPSEC-RI (Figure 1A) and they have estimated relative molar masses of 2.6 and 14.3 kDa, respectively. The higher GGHS molar mass in comparison with GGH was due to sulfate groups incorporation at the GGHS structure. The degree of substitution (DS) by sulfate groups at the GGHS structure was 1.9 for each monosaccharide. Regarding the monosaccharide composition, GGH was composed of mannose and galactose in a 1.8/1 ratio according to ^1^H NMR data (Figure 1B). The 2D-NMR HSQC-DEPT correlation spectra (Figure 1C,D) resembled those reported by Barddal et al.,^44^ suggesting chemical method reproducibility used for polymer synthesis. Notably, a significant shift in signals was observed in the GGHS spectrum compared to the GGH spectrum, indicating that the sulfation process was effective, leading to the insertion of sulfate groups in all or nearly all monosaccharide units. This observation is further supported by the calculated DS described above. The ^13^C/^1^H anomeric signals of both β-d-Man*p* and α-d-Gal*p* units exhibited an upfield shift, attributed to the β-effect resulting from the insertion of sulfate groups at the C-2 position of both units. A similar shift was observed for the glycosidic bond signal corresponding to mannose units at the O-4 position. The glycosidic bond signal shift from 3.723/78.2 in the GGH spectrum to 5.045/77.3 in the GGHS spectrum reflects the insertion of sulfate groups at the O-3 position of these units. Another key feature of the sulfated polysaccharide spectrum is the absence of signals corresponding to free C-6 at δ3.783/62.0 and δ3.641/62.5, which can be detected only in the GGH HSQC spectrum (Figure 1C). The GGHS HSQC spectrum showed β-d-Man*p* H-6/C-6 signals at 4.133/68.5 and 4.065/68.4 which indicates that all mannose units were substituted at O-6 by galactose or sulfate groups. Similarly, the nonreducing terminal galactose residues in the polymer were also 6-*O*-sulfated in the GGHS structure, as evidenced by the H-6/C-6 signals at δ 4.214, 4.178/68.0.

**Figure 1:**
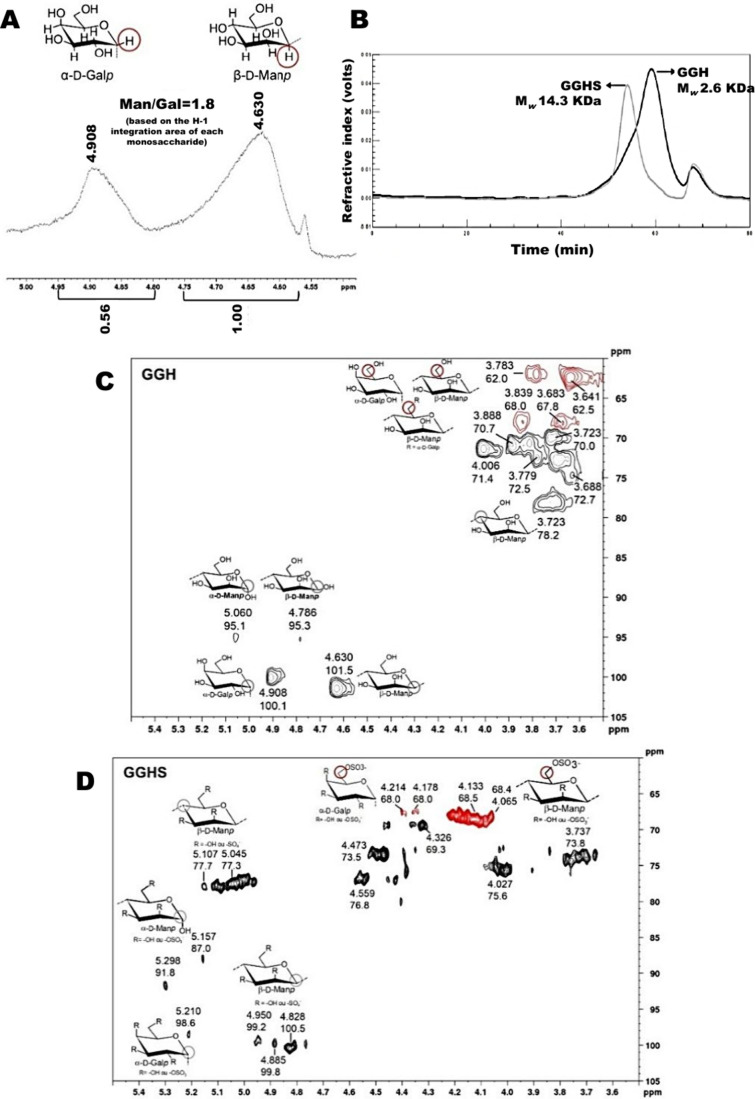
GGH and GGHS characterization. (A) ^1^H NMR spectrum anomeric region of GGH with integration of area of each anomeric. (B) HPSEC-RI elution profile with estimated molar masses of each GGH and GGHS based on a dextran standard curve. (C, D) ^1^H/^13^C signals from 2D-NMR HSQC-Ed spectra of the polysaccharides GGH (C) and GGHS (D). Experiments were performed at 50 °C using a Bruker Avance 400 MHz instrument. The red signals mean inverted ones that correspond to −CH_2_ groups. The monosaccharide drawing structures were GGH and GGHS components, and the circles highlighted some nuclei that are being discussed in the text.

### 3.2 GGH and GGHS were Noncytotoxic, but GGHS Showed Cytostatic Effects on Melanoma Cells

Murine melanoma cells (B16–F10) and nontumorigenic fibroblasts (BALBc/3T3 clone A31) cells from mouse were exposed to GGH and GGHS concentrations ranging from 0.0001 up to 100 μg/mL for 72 h. GGH treatment did not change melanoma and fibroblast viability and proliferation (Figure 2A). However, 100 μg/mL GGHS treatment selectively decreased melanoma cell proliferation without altering their viability (Figure 2A) or inducing cell death (Figure 2B). Accordingly, 100 μg/mL GGHS treatment induced G2 cell cycle arrest (Figure 2C) without leading to cell apoptosis. The ability to sustain cell proliferation signaling is a key characteristic of cancer cells.^56^ Many cancer treatments, including chemotherapeutic agents like Paclitaxel and Vincristine, inhibit cell cycle progression by disrupting tubulin dynamics, preventing mitosis. Plant polysaccharides and seaweed sulfated polysaccharides have been shown to block cell cycle progression of tumor cells, including melanoma.^57^

**Figure 2:**
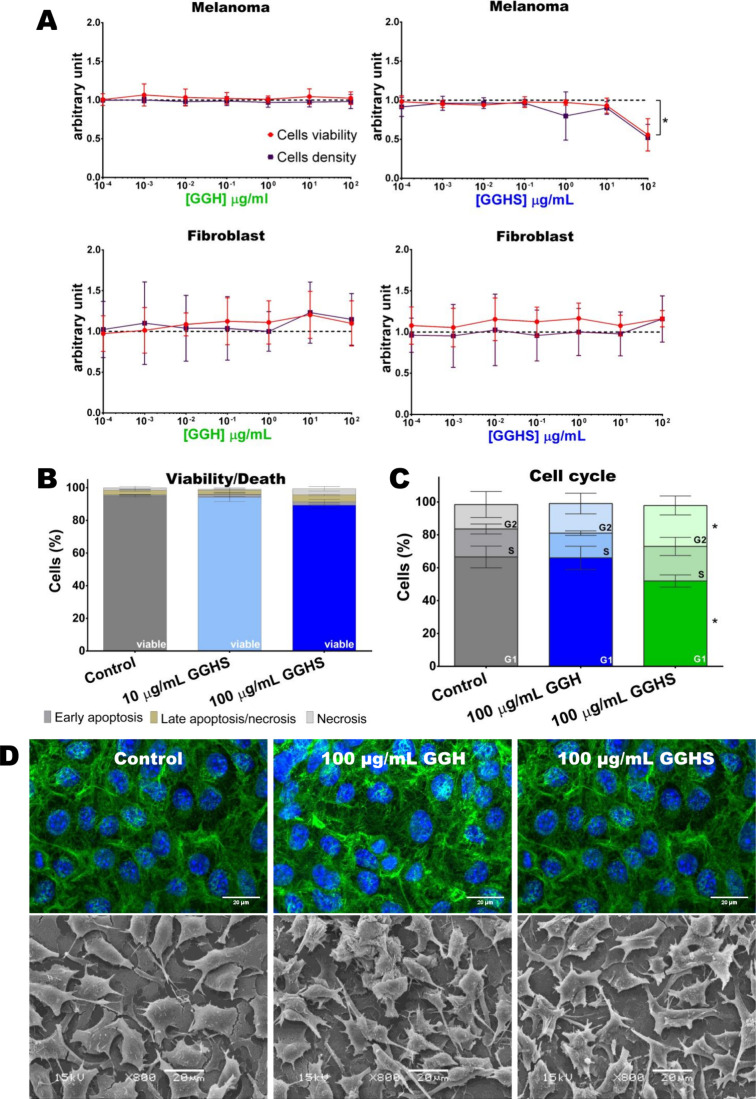
Influence of GGH and GGHS *in vitro* treatment on cells viability, death, cycle, and morphology. (A) B16–F10 melanoma cells and BALBc/3T3 fibroblasts,72 h treated with different concentrations of GGH or GGHS were assay for cell viability (neutral red uptake – red lines) and cell density (crystal violet staining – purple lines). Dashed line represents control. Each dot represents the mean of technical triplicate ± SD of four independent experiments. Data within each assay was analyzed by ordinary one-way ANOVA with Dunnett’s multiple comparison test (*p < 0.05). Data from GGH and GGHS groups are presented separately to enhance clarity and facilitate visualization. (B) Melanoma cells (B16–F10) treated for 72 h with 10 or 100 μg/mL GGHS were analyzed by flow cytometry using Annexin V and PI staining. Stacked bars represent mean with standard deviation, from four independent experiments. Ordinary one-way ANOVA with Dunnett’s multiple comparisons test was performed (*p* < 0.05). (C) Cell cycle determination of melanoma cells (B16–F10) treated for 72 h with 10 or 100 μg/mL GGHS by DNA staining (PI) and analyzed by flow cytometry. Stacked bars represent mean with standard deviation, from four independent experiments. Two-way ANOVA with Dunnett’s multiple comparisons test was performed (*p < 0.05). (D) Representative images of each group. Melanoma cells (B16–F10) were cultured on coverslips, exposed to 100 μg/mL GGH or GGHS for 72 h, fixed with 2% PFA, stained with DAPI (for the nuclei; blue color) and ActinGreen (for actin filaments; green color), and observed by confocal microscopy (upper row) or fixed with 2.5% glutaraldehyde, 1% osmium tetroxide, processed, and imaged by scanning electron microscopy (bottom row). Scale bar: 20 μm.

*Laetiporus sulphureus*-derived sulfated polysaccharides (SPS) exhibit antineoplastic activity against breast cancer cells, mediated through the induction of cell cycle arrest. Mechanistically, SPS treatment resulted in cell cycle arrest at the G0/G1 phase, characterized by a downregulation of cyclin-dependent kinase 4 (CDK4) and cyclin D1, concomitant with an upregulation of p21 protein expression.^58^ Given the established role of cyclin-dependent kinases (CDKs) and cyclins in regulating cell cycle progression,^59,60^ these findings suggest that SPS exerts its effect by modulating the expression of key cell cycle regulatory proteins, leading to G0/G1 phase arrest through the specific reduction of CDK4 and cyclin D1 and elevation of p21. Considering that the GGHS arrested the cell cycle in the G2 phase (Figure 2C) and that it may be attributed to the downregulation of CDK1, p-CDK1, and cyclin B1 ^60,61^ targeting these molecules in further experiments could explain the interaction between GGHS and the effect in melanoma cell cycle arrest.

Galactomannan-type plant polysaccharides were described to induce selective cytotoxicity in tumor cells such as those from *Sesbania cannabina* that showed concentration-dependent cytotoxicity against lung, breast, liver, and cervical cancer cells. At 100 μg/mL, the 4.9 kDa galactomannan reduced tumor cell viability by 40–60% via caspase-12 activation.^62,63^ Although cytotoxic chemotherapy agents were employed as the main treatment for melanoma for over 30 years they are now being replaced by targeted therapies, immunotherapies, or a combination of these approaches due to their commonly nontarget specificity and consequently associated adverse effects.^64,65^ Therefore, to avoid adverse effects, we further investigated 100 μg/mL GGH and GGHS, the noncytotoxic concentration on the following analyses. Also, the chosen concentration is the concentration largely studied for polysaccharides that present antitumoral activities.^16,17,57,62^

Cells morphological analysis provides insights into the cellular and physiological state since some intracellular molecular processes such as the actin-polymerization, adhesion pattern to an extracellular matrix, and membrane tension can be evaluated.^66^ Representative areas were imaged by confocal microscopy and SEM (Figure 2D), and no differences in cell morphology were observed when comparing control with GGH or GGHS treated cells cultivated directly in the glass slides. However, when cells were cultivated over Matrigel and then treated, slightly different patterns were observed. Cells treated with 100 μg/mL GGH were more spread than control cells, and when treated with 100 μg/mL GGHS colony-like formation regions were observed (Figure 3D). Interaction between sulfated extracellular matrix components with proteins and other compounds were reported to be related to differential adhesion profile and invasiveness.^63,67^ Therefore, the observed pattern in GGHS treated cells could be induced by some interaction between the sulfated polysaccharide with Matrigel.

**Figure 3:**
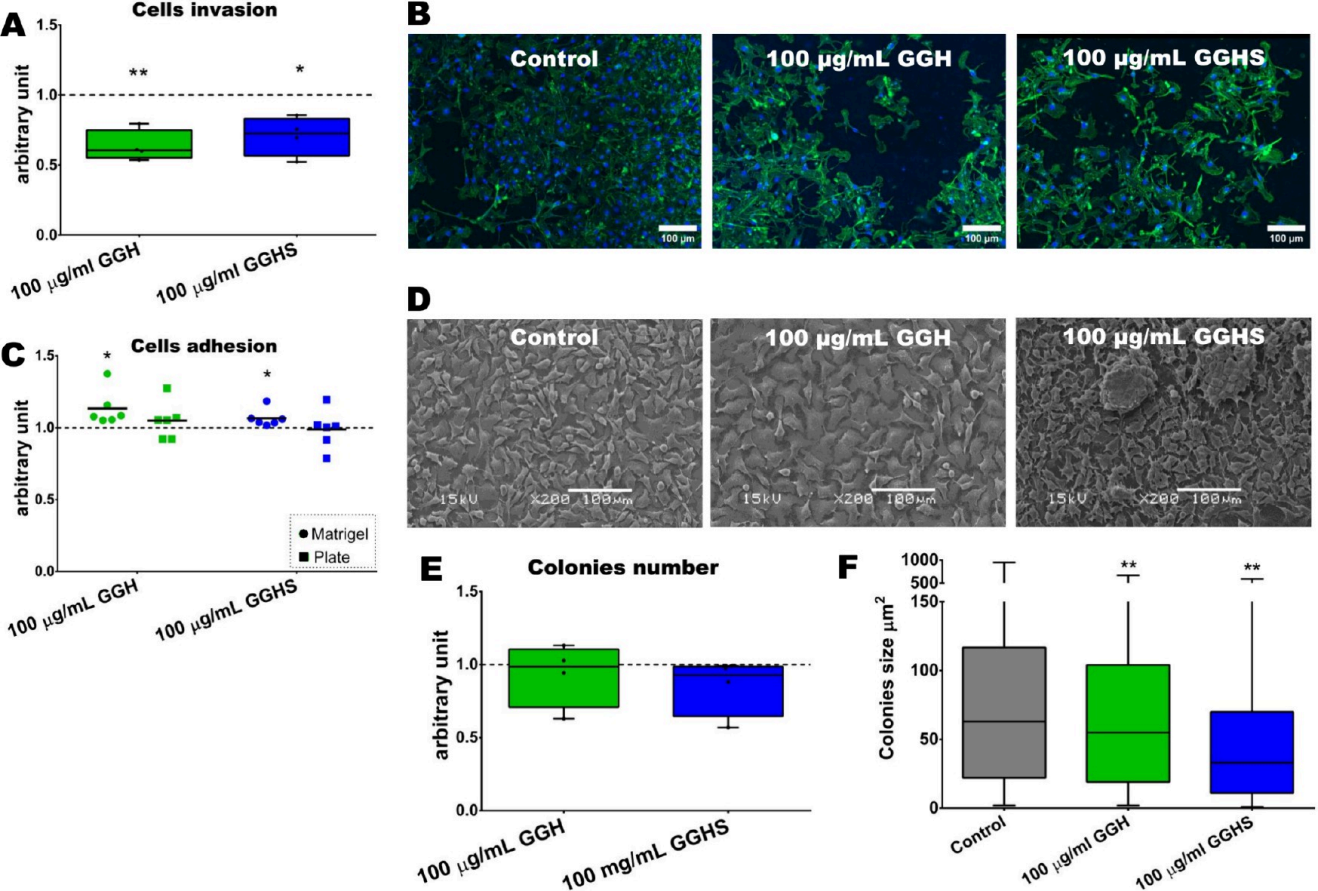
*In vitro* analysis of melanoma metastatic parameters. Melanoma cells (B16–F10) were treated with 100 μg/mL GGH or GGHS for 72 h. (A, B) Invasion assay on Matrigel-coated inserts was performed after B16–F10 cells pretreatment. (A) Nuclei number analyses per mm^2^ of the whole insert, normalized and relative to control group. Data are presented as box with min and max from three independent experiments. Group comparisons were performed using Ordinary one-way ANOVA with Dunnett’s multiple comparison test (*p < 0.05; **p < 0.01). (B) Representative images of each group. Nuclei were stained with DAPI (blue) and actin filaments stained with ActinGreen (green). Scale bar, 100 μm. (C) Analyses of cells adhesion to plastic or Matrigel. Each dot represents the mean of technical triplicates, of six independent experiments. Group comparisons were performed using Welch’s *t* test (*p < 0.05; **p < 0.01). (D) Representative images of each group. Melanoma cells (B16–F10) were cultured on Matrigel -coated coverslips, exposed to 100 μg/mL GGH or GGHS for 72 h, fixed with 2.5% glutaraldehyde, 1% osmium tetroxide, and processed and imaged by scanning electron microscopy. Scale bar - 100 μm. (E, F) Colony formation was analyzed after B16–F10 cells plating at low confluence (400 cells/well) and 96 h of treatment in the presence of 100 μg/mL GGH or GGHS, or without treatment (control). Data are presented as box with min and max from three independent experiments. (E) Colony numbers and (F) colony sizes were compared between groups using ordinary one-way ANOVA with Dunnett’s multiple comparison test (**p < 0.01). (A, C, and E) Dashed line represents normalized data from each respective control group.

### 3.3 GGH and GGHS Alter Malignancy Parameters of Melanoma Cells

For a tumor cell to give rise to metastatic foci, it must degrade the extracellular matrix, migrate, and be able to form new colonies in other tissues.^68^ Therefore, we analyzed GGH and GGHS treatments regarding malignancy-related parameters. Our results demonstrate a reduced invasive capacity of melanoma cells by 36% and 29%, when cells were pretreated with 100 μg/mL GGH or GGHS, respectively (Figure 3A, B). These results are highly relevant, as without altering cells viability, the treatments were able to reduce a characteristic that is crucial for melanoma malignancy and tumor progression.^69,70^ The galactomannan extracted from pomegranate (PSP001), at 100 μg/mL concentration, was shown to reduce melanoma cell invasiveness by 80–90% both on murine (B16–F10) and human (A375) melanoma cells. However, this effect was observed only during simultaneous treatment, meaning that the cells were exposed to the compound solely during the invasion period.^27^ In the experiments conducted in our study, the cells were pretreated for 72 h, and after treatment, they were detached and replated over Matrigel. During the invasion phase, the cells were no longer exposed to the compound, suggesting a potential reprogramming effect on the cancer cells.

Sulfated fucans with a lower anticoagulant activity compared to heparin, FucSulf1 and FucSulf2 at 100 μg/mL derived from echinoderms, exhibit direct antitumor effects by interfering with key cellular mechanisms. They promoted the endocytosis of neuropilin-1 and β1-integrin, induced degradation of focal adhesion kinase (FAK), and disrupted fibronectin-mediated adhesion. These actions collectively impaired tumor cell adhesion, proliferation, migration, invasion, and spreading by weakening adhesion-dependent signaling pathways.^71^ These targets could be further explored to better understand the GGHS affected pathways that result in melanoma invasiveness reduction.

Extracellular matrix (ECM) degradation is essential for cellular invasion, and matrix metalloproteinases (MMPs) play an important role in this process.^70,72,73^ A sulfated polysaccharide from seaweed *Gelidium crinale* can downregulate the expression and activity of MMP-9, thereby reducing cellular invasiveness.^74,75^

Cells adhesion is another important factor in metastasis. Both GGH and GGHS treatment increased cells adhesion when cells were cultivated in Matrigel-coated coverslips but had no effect on cells cultivated directly on the glass (Figure 3C, D). Probably both treatments interact with the extracellular matrix, since the results were observed on the Matrigel-coated glass. Although no specific adhesion effects were described by galactomannan on melanoma (B16–F10) cells, this effect was previously observed with other polysaccharides such as arabinogalactans (78 kDa and 12 kDa) from *Prunus domestica*.^76^ Concerning sulfated polysaccharides, low molecular weight heparins (LMWHs), such as enoxaparin and tinzaparin, effectively inhibit VLA-4-mediated cell binding, primarily through interactions with the integrin α-chain.^77,78^ It is noteworthy that VLA-4 plays a critical role in the metastatic dissemination of melanoma cells.^79,80^

The clonogenic assay revealed that treatment with GGH or GGHS did not affect the number of colonies formed by melanoma (B16–F10) cells but reduced the colonies area by 12% and 48%, respectively, when compared to the control (Figure 3E, F). This data corroborate the cell cycle arrest observed in the GGHS treated group (Figure 2C); the diminished colonies size could be due to the cytostatic effect observed in the cell cycle assay. Treatment with 100 μg/mL GGHS resulted in greater homogeneity in colony size, suggesting reduced variability in the cells phenotype.

### 3.4 GGH and GGHS are Galectin-3 Ligands

Several cellular processes are important for the tumor progression dynamics. In the context of melanoma, changes in the tumor microenvironment, such as overexpression and secretion of galectin-3, are essential for increasing the stimulation and survival of melanoma cells, generating an increase in angiogenesis, cells-extracellular matrix interaction, vascular dissemination, and extravasation.^81–83^ The concentration of Gal-3 is elevated in the circulation of melanoma patients and those with metastasis have even higher levels.^84^ Interestingly, Gal-3 has been described as a target of polysaccharides with antitumor effects, including those that inhibit melanoma development and metastasis.^52,85,86^

Considering that DAVANAT effects were suggested to be mediated by galectin-1 and -3 binding;^31,32^ that GGH is a DAVANAT-mimetic; and that galectin-3 is overexpressed in melanomas,^87^ we performed galectin-3 binding assays with both GGH and its sulfated derivative GGHS. Lactose was used as a positive binding control and its behavior was compared with GGH and GGHS. Although with lower magnitude than lactose, both GGH and GGHS were observed to be galectin-3 ligands, determined by an increase in the adsorbed mass [(ng)/cm^2^] (Figure 4). GGHS presented a greater adsorbed mass when compared to GGH, likely due to its greater molar mass. Although lactose molar mass is 7 times smaller than GGH molar mass, the high adsorbed quantity could be due to its better binding capacity compared to the tested polysaccharides.

**Figure 4:**
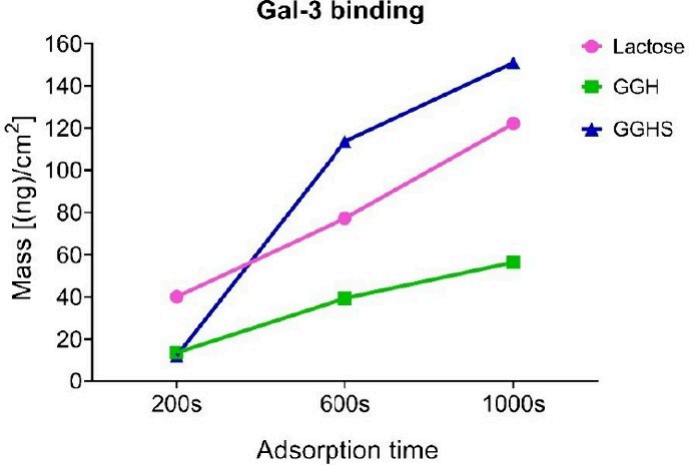
Galectin-3 binding assay by QCM-D (quartz crystal microbalance with dissipation monitoring) analysis. GGH and GGHS (1000 μg/mL) were subjected to binding assays to galectin-3 that was bound to the balance sensor. Lactose (10 mg/mL) was used as a positive binding control. Graph displaying three measured adsorption time points (200, 600, and 1000 s).

Being galectin-3, a β-galactoside-binding lectin overexpressed in melanoma, that is a marker of progression in melanocytic lesions and prognosis in primary melanoma patients,^87^ galectin-3 binding agents can generate a process of blocking of its known pro-tumor action.^88–90^

Galectin-1 (Gal-1) is known to be deeply involved in the initiation, amplification, and resolution of inflammatory responses. In melanoma, Gal-1 binding to Gal-1 ligands on immune and endothelial cells thus influencing melanoma development through inhibition of antitumor immune responses and angiogenesis.^91,92^ DAVANAT, similar to GGH, binds to a noncanonical site on Gal-1, distinct from the traditional carbohydrate recognition domain (CRD). This alternative binding site spans a substantial area across the dimer interface, situated on the side of the protein opposite the lactose-binding site. Quantitative analyses indicated that Gal-1 binds with DAVANAT with significantly higher affinity compared to lactose. Notably, the CRD remains accessible, as lactose can still bind without apparent loss in affinity. As expected, GGH bound to Gal-1 (Figure S1). Interestingly, GGHS bound to Gal-1 in even more pronounced form than GGH, as observed with Gal-3 and this could be related with sulfation.

It has been described that sulfated polysaccharides bind to Gal-3 with higher affinity than nonsulfated ones.^93^ Using NMR spectroscopy and molecular dynamics simulations, it was demonstrated that keratan sulfate (KS) binds to Gal-3 with a significantly higher affinity compared to its desulfated counterpart. The enhanced binding could be attributed to the presence of negatively charged sulfate groups, which are likely to promote electrostatic interactions with positively charged residues on the galectin surface. The sulfation is critical in galectin-carbohydrate recognition. and the binding mechanism of sulfated polysaccharides seems to involve other than the canonical one, which could explain the higher galectin-binding capacity.^93^ The sulfated polysaccharides have also been described as classical Gal-ligands. Heparin and their chemically modified and low molecular weight derivatives bind to the Gal-3 CRD domain and inhibit Gal-3-mediated melanoma cell adhesion, invasion, and angiogenesis.^86,94^

Taking together these results suggest that both GGH and GGHS were Gal-3 and Gal-1 ligands that can explain at least in part the *in vitro* evidence of GGH and GGHS attenuation of melanoma cells malignant features, leading us to carry out further *in vivo* experiments to determine its effects on melanoma-bearing mice.

### 3.5 GGH but Not GGHS Presented *In Vivo* Antitumor Effects

The *in vivo* experiments showed that 5 mg/kg of GGH significantly impaired solid tumor growth. In contrast, no statistically significant differences were detected in the solid tumors of GGHS-treated animals (Figure 5A,B). Microscopic qualitative analyses of histological sections demonstrated that the GGH-treated group presented relatively smaller necrotic areas than the control (Figure 5B). Although not statistically different, when lung colonization was analyzed in GGH and GGHS treated animals, a smaller area was observed (30.8% and 25.6%, respectively) compared to the control group (Figure 5C,D). Adverse effects were not observed in the treated animals with both treatments (Supplementary Tables 1–5), supporting their potential use as a treatment.

**Figure 5:**
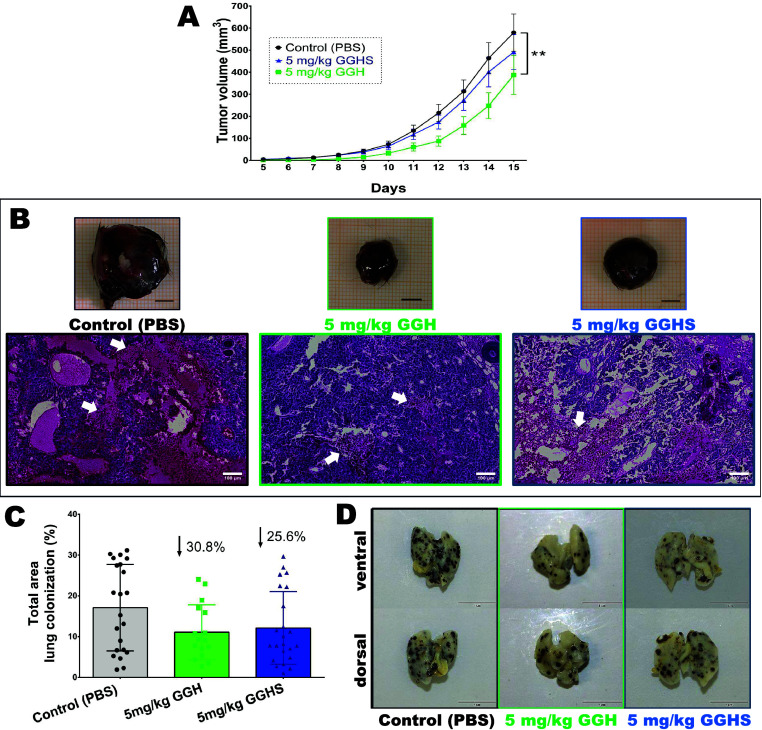
Solid tumor progression and lung metastasis model analyses. (A) Tumor volume (mm^3^) over time (Control-PBS *n* = 15; 5 mg/kg GGH *n* = 11; 5 mg/kg GGHS *n* = 16, male mice). Two-way ANOVA with Dunnett’s multiple comparisons test was performed (**p < 0.01). Data are presented as mean with standard error of the mean. (B) Macroscopic images representative of tumor size from each group (scale bars - 5 mm). Representative histological sections from each group (scale bars -100 μm). White arrows indicate necrotic areas. (C) Percentage of metastatic foci summed from both lungs views (ventral and dorsal) (Control PBS *n* = 15; GGH 5 mg/kg *n* = 11; GGHS 5 mg/kg *n* = 16, male mice). Bars represent mean with standard deviation. Ordinary one-way ANOVA with Dunnett’s multiple comparisons test was performed (*p* < 0.05). (D) Representative images from lung ventral (first row) and dorsal (second row) views from each group (scale bars - 1 cm).

It is known that DAVANAT (51 kDa), which is also obtained from guar gum and presents highly structural similarity to GGH, leads to CD8+ tumor-infiltrating lymphocyte cytotoxicity through Gal-1 linkage.^95,96^ It also inhibited colon, mammary, and melanoma tumor growth in preclinical experiments. When tested in humans (clinical phases I and II) the DAVANAT, at 280 mg/m^2^/day, was nontoxic, increased the longevity of the patients and reduced colon tumor growth.^31^ Therefore, the tumor growth impairment observed by GGH treatment, together with its effects on tumor necrotic areas, agrees with literature results about the DAVANAT antimelanoma effect.

The galactomannan PSP001 (110 kDa) from *Punica granatum* has the same repeated structure of GGH although with a higher molar mass. Treatment with 200 mg/kg PSP001 significantly reduced lung metastases in B16–F10 melanoma-bearing mice treated. When combined with vincristine, PSP001 also decreased metastatic nodules, suggesting its potential as an adjuvant therapy.^26^

The lack of *in vivo* efficacy observed for the sulfated polysaccharide, despite its *in vitro* activity, may be explained in part by its pharmacokinetic behavior and biodistribution after administration. A study evaluating the pharmacokinetics and tissue distribution of fluorescently labeled carrageenan (a sulfated galactan) showed that after oral administration in mice, the compound reached peak plasma levels at approximately 3 h, exhibited a mean residence time of 36.6 h, and had a clearance rate of 0.1 L/h/kg. Notably, the compound accumulated primarily in the liver and kidneys, with the majority excreted in the feces and only a minor fraction excreted in the urine, suggesting limited systemic absorption and possible metabolic transformation in hepatic or intestinal compartments. Histological analyses on kidney and liver of the treated mice suggested potential chemical modification or degradation *in vivo*.^97^ These findings highlight the possibility that the polysaccharide may be rapidly metabolized, structurally altered, or sequestered in nontarget tissues, which could impair its availability and activity at the intended site of action. Therefore, such pharmacokinetic limitations must be considered when interpreting the GGHS absence of biological effects *in vivo*, despite promising *in vitro* data.

In addition to their pharmacokinetic properties, it is important to emphasize that sulfated polysaccharides may exert biological effects through mechanisms distinct from those of their nonsulfated polysaccharides. While some sulfated polysaccharides are known to inhibit Gal-3 activity (e.g.,^94^), their *in vivo* effects are often mediated by interactions with a broader range of extracellular matrix (ECM) components and signaling pathways. It was reported that VLA-4 integrin binding of murine or human melanoma cells to VCAM-1 can be inhibited by heparin and low molecular heparin derivatives, and this effect seems to be structure-dependent.^78–80,98^ The high expression levels of VLA-4 integrin in melanoma cells can partly contribute to this malignancy by driving contact formation with the endothelium at distant sites, binding to the ligand VCAM-1. Nonanticoagulant heparins can inhibit P- and L-selectin and heparanase activity in the course of metastasis.^98^ The P- and L-selectins also facilitate the metastasis of cancer cells by mediating interactions with platelets, endothelium, and leukocytes.

Heparanase is also an important target of sulfated polysaccharides, such as heparin. Heparanase degrades heparan sulfate of extracellular matrix, thereby promoting tumor invasion and metastasis.^98^ Beyond heparin and its derivatives, several other anionic molecules inhibit heparanases and can diminish metastasis, presumably because it mimics the tumor cell surface ligands, which contain negatively charged sialic acids and tyrosine sulfate residues.^99,100^ The commercial laminarin (β-glucan) chemically sulfated mimics the inhibitory effect of heparin on heparanase activity and on melanoma metastasis. In contrast, the nonsulfated laminarin failed to inhibit either heparanase or melanoma tumor metastasis, indicating that sulfate groups play a decisive role in the antimetastatic effect. It is noteworthy that beyond natural sulfated polysaccharides, synthetic polyanions may be applied as heparanase inhibitors and lead to tumor metastasis inhibition such as phosphorothioate oligodeoxynucleotides.^101^

The sulfated polysaccharides could also inhibit metastasis by decrease the matrix metalloproteinases (MMPs), particularly MMP-2 and MMP-9 that degrade extracellular matrix facilitating cell migration, extravasation, and intravasation during the metastasis steps.^74,102^

As observed in Figure 5A, GGHS did not inhibit primary tumor development, but a reduced area of lung metastasis is suggested (Figure 5C). This effect could be related to its action on P-selectins, which impair tumor cell interactions with platelets and the endothelium; on heparanase, reducing tumor cell intravasation at secondary sites; or on the expression and/or activity of MMPs, rather than being primarily associated with Gal-3 binding.

In contrast, GGH may act through a different mechanism involving the inhibition of both Gal-3 and Gal-1, which could explain its effects in the two tested models. These findings suggest that the observed reduction in metastatic foci is linked to distinct molecular targets in each treatment. Of course, we cannot overlook that, in addition to potentially distinct mechanisms of action and molecular targets, GGH and GGHS may also differ in their pharmacokinetic and pharmacodynamic properties, which could contribute to the differences observed in their *in vivo* efficacy. A clear limitation of our study is the lack of direct *in vivo* experimental evidence to support these assumptions. We recognize the importance of conducting more comprehensive *in vivo* investigations in the future, including assessment of GGH and GGHS stability within the biological system, biodistribution analyses, as well as the use of galectin knockout mice. These studies will be crucial for elucidating the mechanisms underlying the differential therapeutic responses of GGH and GGHS.

### 3.6 GGH and GGHS Modulated Tumor Microenvironment Immune Cells

T lymphocytes infiltration into the tumor is extremely relevant for melanoma development and increasing patient survival.^103,104^ Therefore, ensuring a considerable presence of these cells during disease progression is a major focus of study to guide new treatment approaches for melanoma.^105^ Even though the Jurkat cells are an immortalized T lymphocyte cell line obtained from a T-cell leukemia patient, it has been extensively employed and validated as a model of T-cell activation, proliferation, and intracellular signaling mechanism.^106^ Thus, Jurkat cell proliferation was evaluated after 100 μg/mL of GGH and GGHS treatment. It was observed that neither GGH nor GGHS altered lymphocytes (Jurkat cells) proliferation profile (Figure 6A).

**Figure 6:**
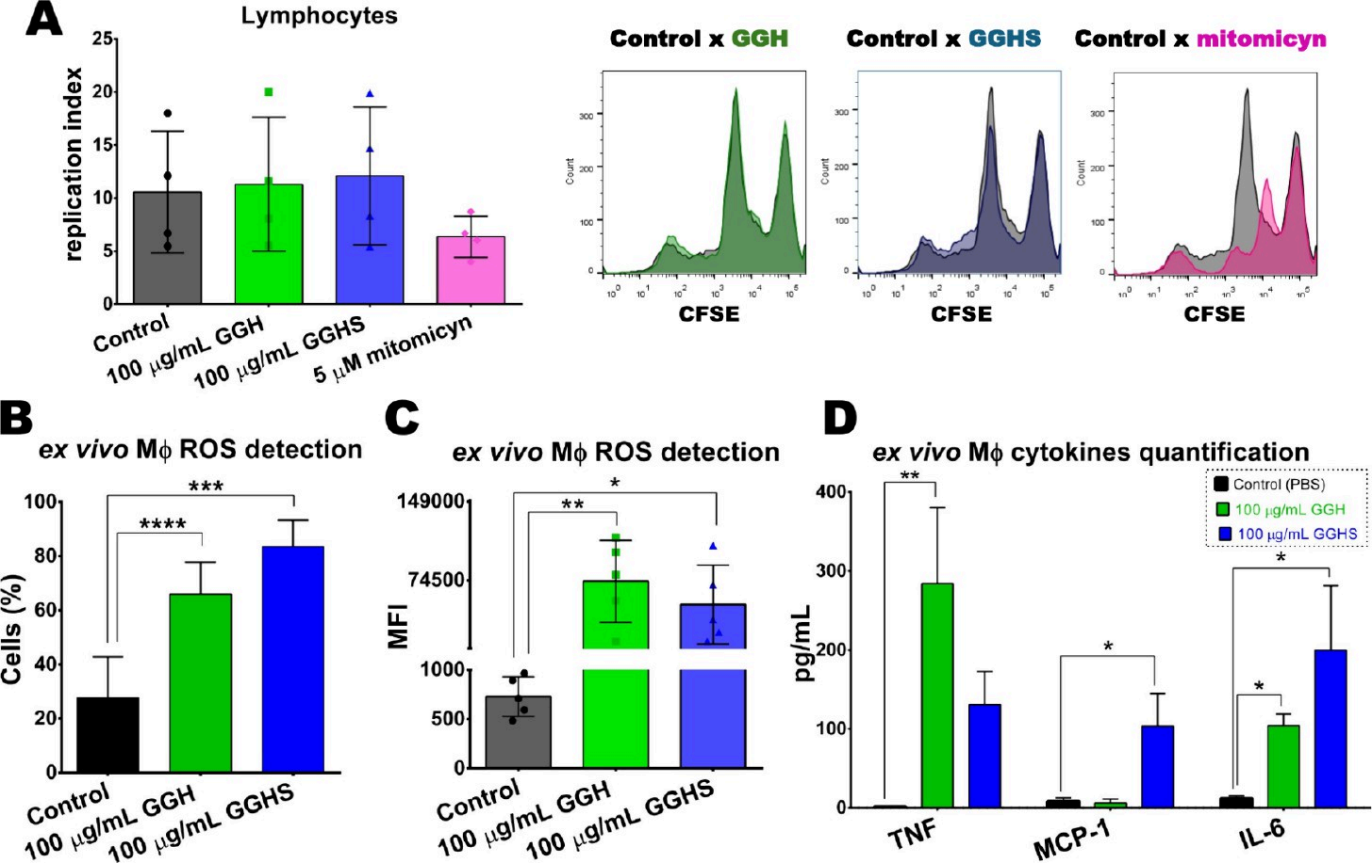
GGH and GGHS effects on lymphocytes and macrophages. (A) T-cell replication index (number of divided cells per number of cells that entered division). Histograms of 100 μg/mL GGH (green), 100 μg/mL GGHS (blue), and 5 μM Mitomycin (pink) treated cells compared to the control (RPMI 1640 medium–gray). (B, C) *Ex vivo* Reactive Oxygen Species (ROS) detection in peritoneal macrophages from tumor-bearing mice. (B) Percentage of cells in the population with a larger and more granular profile in each group. (C) Median fluorescence intensity (MFI) of the analyzed population was obtained using the DCF probe. (D) Quantification of cytokines released by peritoneal-derived macrophages. Statistical analyses were performed using ordinary one-way ANOVA with Dunnett’s multiple comparisons test (*p < 0.05; **p < 0.01; ***p < 0.001; ****p < 0.0001).

The immunostimulatory effects of galactomannans, particularly the sulfated ones, on T lymphocytes remain poorly understood. This activity is closely linked to the molecular structure as factors like structural differences, molecular weight, and composition influence their immune potential. A study showed that PAC-I, a polysaccharide from *Aloe vera* with a β-(1 → 4)-mannose backbone like GGH and GGHS, induced T lymphocyte proliferation in BALB/c mice in a concentration-dependent manner (100–500 μg/mL).^107^ Conversely, a galactomannan from *Ceratonia siliqua* with the same structure as GGH but higher molar mass (50–100 kDa) did not promote T-cell proliferation *in vivo* (25 μg/mL).^108^

Although GGH and GGHS did not change *in vitro* Jurkat T lymphocyte proliferation, T-cell infiltration into tumors depends on other factors, such as cytokines and vascular adhesion.^104,109^ These compounds may still contribute to T-cell recruitment and activation, potentially increasing tumor and bloodstream lymphocyte infiltration *in vivo*. Additionally, they may have immunostimulatory effects on other immune cells such as macrophages.

Macrophages are key immune defense cells that play a crucial role in melanoma progression, depending on their profile.^110–112^ M1 macrophages produce high levels of pro-inflammatory cytokines such as TNFα and IL-6, and reactive oxygen species (ROS) as part of their inflammatory response,^113^ and upregulate antigen-presenting molecules (MHC II, CD80, and CD860)^114^ that grant a strong antitumor potential. In contrast, M2 macrophages promote tumor progression by producing and releasing immunosuppressive cytokine such as TGFβ, IL4 and IL13, and regulating molecules such as CD206 and CD163 that facilitate tumor growth and metastasis.^115,116^ Tumor associated macrophages (TAMs) are commonly associated with the M2-like profile, and the balance between M1/M2 macrophage polarization settings is linked to disease progression or resolution.^114^

In this context, ROS levels and cytokine production could potentially indicate the treatment outcome. Therefore, to determine GGH and GGHS influence on macrophages from tumor-bearing mice, these molecules production was accessed. It was observed that, compared to the control, both compounds promoted macrophage activation, increasing ROS-producing cells number as well as the amount of ROS produced (Figure 6B, C). Similar polysaccharides have shown comparable effects on macrophages. A *Coffea arabica* polysaccharide rich in galactose and mannose increased ROS production in BALB/c peritoneal macrophages at 100 μg/mL for 24h.^117^ Likewise, a sulfated polysaccharide from *Caulerpa cupressoides* with galactose, mannose, and xylose significantly enhanced ROS production in RAW 264.7 macrophages at 800 μg/mL after 24 h of exposure.^20^

As mentioned previously, pro-inflammatory cytokines are mainly produced by activated macrophages and mediate immune responses.^114,118^ Our results of cytokine analysis in the macrophage culture supernatant revealed a significant increase in TNF levels in the GGH group, but lower levels were detected in the GGHS group (Figure 6D). Guar gum and its derivatives (C-glycosylated and sulfated) were demonstrated to induce TNFα release by the macrophage cell line RAW 264.7. Likewise, TNFα levels were lower when comparing the native molecule with the sulfated derivative.^30^ MCP-1 level was increased by GGHS treatment but not by GGH. Both treatments significantly increased IL-6, but again, lower levels were detected in the GGHS group (Figure 6D). In the melanoma context, MCP-1 and IL-6 play a dual role, both inhibiting tumor progression and promoting metastasis, depending on the tumor microenvironment.^8,119,120^ Taken together, these results indicate that both GGH and GGHS promote macrophage polarization toward an M1 phenotype. GGH showed a stronger effect on M1 polarization than GGHS, likely due to its significant TNF increase, which promotes an antitumoral macrophage profile. In addition to these results, bone marrow derived macrophages from healthy mice were treated with both molecules and an increase in CD80+ and CD86+ cells was observed in the GGH treated group (Supporting Information, Figure S2).

Although we did not currently investigate the details about pathways involved in macrophage modulation from GGH and GGHS treated melanoma-bearing mice (both *in vivo* and *ex vivo*), their Gal-3 and Gal-1 binding could be involved. Gal-3 is upregulated in tumor-associated macrophages (TAMs) and tends to support the M2-like, immunosuppressive phenotype. The use of Gal-3 inhibitors in lung cancer models resulted in decreased M2-like tumor-associated macrophages (TAMs) and increased markers associated with M1 macrophages which is generally more antitumoral.^121^ Galectin-1 is also highly expressed in TME, and it is related with overexpression of chemokines such as CCL20 in TAMs via activation of the PI3K/AKT/NF-κB. Also, Gal-1 induces the expression of immune checkpoint molecules such as PD-L1 in TAMs, contributing to T-cell dysfunction.^122^ Thus, both Gal-1 and Gal-3 are directly related to the immunosuppressive environment of the tumor microenvironment participating in crosstalk between tumor and immune cells. Targeting Gal-3 and Gal-1 has shown promise in reprogramming TAMs toward a pro-inflammatory, antitumor phenotype.^123^ and can enhance the efficacy of cancer immunotherapies as already described in melanoma.^124^

Knowing that the tumor microenvironment milieu provides immunostimulatory or immunoinhibitory stimuli and has been implicated as target to improve antimelanoma drug efficacy,^125^ we next investigated GGH and GGHS effects on cytokine production and lymphocytes population in the tumors and lungs of melanoma-bearing animals.

### 3.7 Cytokine and T-Cell Immunophenotyping Analysis in Solid Tumor and Lung Experimental Metastasis

In the melanoma context, it is long known that the infiltration of immune system cells into tumor and the presence of cytokines play a crucial role in disease progression.^11^

Tissue cytokine quantification showed no significant differences between treatment groups in either the tumor (Figure 7A) or the lungs (Figure 7C). Lower levels of TNF and IL-6 were observed from tissue when compared with levels released by macrophages from melanoma-bearing mice (Figure 6D). *In vitro*, it was demonstrated that synthetically prepared isomeric pentasaccharides representing fragments of *Aspergillus fumigatus* cell-wall galactomannan and containing β-(1 → 5)-linked tetra galactofuranoside induced and increase release of TNFα and IL-6 by RAW 264.7 macrophages after 24 h of exposure, with lower levels detected after 48 h.^126^ Probably, the results obtained here are likely due to differences in compound exposure, as *ex vivo* cells had direct contact to GGH and GGHS, whereas *in vivo* distribution was systemic.

**Figure 7:**
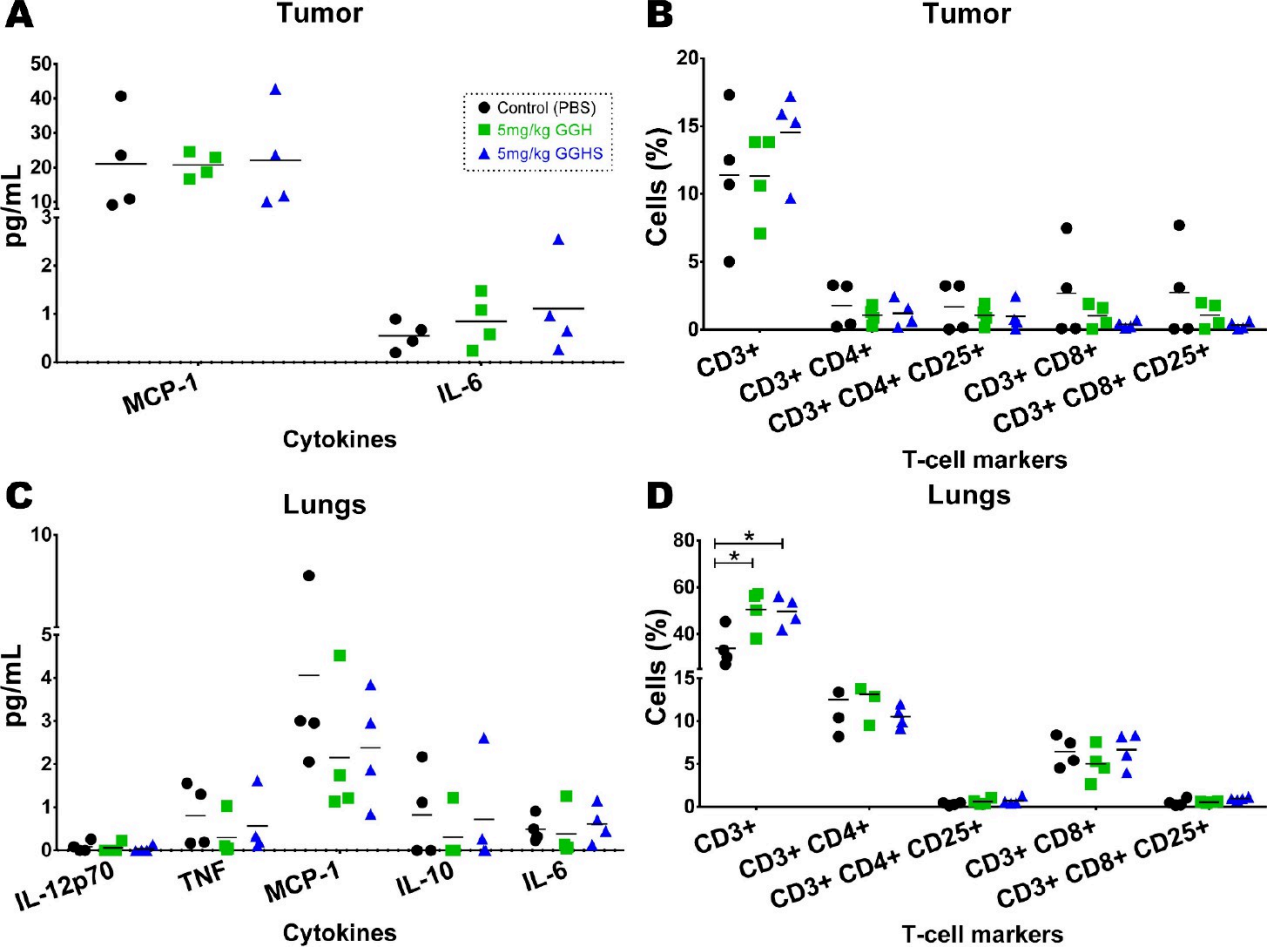
Tissue cytokine analysis and T-cell immunophenotyping of *in vivo* tumor-bearing treated mice. (A) Cytokines and (B) percentage of labeled T-lymphocytes within the tumor based on antibody profiling. (C) Cytokines and (D) percentage of labeled lymphocytes in lungs based on antibody profiling. (A–D) Data are presented as individual values with mean (*n* = 4). Statistical analysis was performed using the one-way ANOVA with Dunnett’s multiple comparisons test (*p < 0.05).

T-cell immunophenotyping assay revealed a significant increase in the number of CD3+ T lymphocytes in the metastatic lung (Figure 7D) but not in the primary tumor (Figure 7B). Treatment with GGH and GGHS significantly increased lymphocyte (CD3+ cells) infiltration in lung metastatic foci, even though the *in vitro* assays showed no effect on Jurkat lymphocytes (Figure 6A). *In vitro* assays present inherent limitations, as they do not fully recapitulate the complex immunological and microenvironment context of *in vivo* systems. This likely explains the observed difference between the results. Nevertheless, the observed increase in CD3+ T-cells in pulmonary metastatic nodules could also be a consequence of cell recruitment by chemokines rather than proliferation. T-cells activation and expansion *in vivo* are closely related to the cytokine profile. In the case of tumor infiltrating immune cells, they are specially recruited by chemokines in different types of cancers,^127^ including in melanoma,^128,129^ and can be targeted to modulate and promote tumor immunosurveillance.^130^

More important, the profile of infiltrated T-cell subsets can shed light into the disease outcome.^131–133^ Therefore, at the experiment end point we accessed tissue infiltrated helper (CD4+), cytotoxic (CD8+), and regulatory (CD25+) T-cell subsets, based on T-cell classification. The gating strategy used efficiently enabled infiltrated lymphocyte subpopulation identification and quantification within the tumor and lung. However, no statistical differences were observed among their expressions throughout the tested groups (Figure 7B,D). It is noteworthy that the tumor microenvironment is extremely dynamic. Therefore, future *in vivo* studies would be necessary to determine the mechanism underlying the increase in CD3+ T-cells following GGH and GGHS treatment, particularly to determine whether this effect is regulated by chemokines signaling. Additionally, it is important to investigate how GGH and GGHS treatments influence on activity and dynamics of specific T-cells subsets over the course of therapy to fully understand the observed effects..

## 4 Conclusion

GGH and GGHS were not cytotoxic, and GGHS was selectively cytostatic against melanoma cells. As expected, some common effects resemble DAVANAT effects, and the sulfate insertion changes some of them. Although both GGH and GGHS showed similar antimelanoma effects when tested *in vitro*, through reduction invasion and clonogenic capacity, GGH was more effective to impair primary melanoma tumors development. Even though no significant changes in melanoma foci were observed in both GGH and GGHS treated groups, the tumor microenvironment analysis showed that both polysaccharides induced lymphocyte infiltration in the lungs of melanoma-bearing mice. *Ex vivo* results using bone marrow derived macrophages also supported GGH and GGHS effects in modulating their phenotype toward pro-inflammatory macrophages. The observed systemic effects of GGH and GGHS could be, at least in part, explained by their galectin-3 binding capacity. It is noteworthy to highlight that even though GGHS did not showed significant advantage compared with GGH, which is a DAVANAT mimetic, previous research showed that GGHS had anticoagulant effect which could add another important therapeutic benefit helping in preventing the cancer associate thromboembolism in future clinical applications.

The use of GGH, GGHS, low-anticoagulant heparins, and sulfated polysaccharides such as fucans hold strong promise as adjuvant therapies in cancer by targeting metastasis and tumor growth through noncytotoxic, antiadhesive, and anti-invasive mechanisms, especially valuable for patients at risk of bleeding or those undergoing conventional therapies. Further clinical studies are warranted to confirm safety and efficacy in humans.

## Supplementary Material



## Notes

Organ and tumor weight of the animals; Blood tests: biochemical parameters erythrocyte, leukocyte, and platelet series analyses; Galectin-1 binding assay; BMDM M1 polarization assay (PDF)
