# Supplementary material for: Davanat-Mimetic Galactomannan and Its Sulfated Derivative: Structure and Antitumor Effects against Melanoma
Source: Biomacromolecules. 2025 Aug 21;26(9):5614–32. doi: 10.1021/acs.biomac.5c00290 (PMC12421689; doi:10.1021/acs.biomac.5c00290)
Supplement: Supplementary file 1 [file bm5c00290_si_001.pdf]

Supporting Information

DAVANAT®-MIMETIC GALACTOMANNAN AND ITS SULFATED DERIVATIVE: STRUCTURE AND ANTITUMOR EFFECTS AGAINST MELANOMA

Odair Braz Júnior.[1‡], Aline Miranda Cristal[1‡], Daniel de Lima Bellan[1], Gustavo Rodrigues Rossi[1], Stellee Marcela Petris Biscaia[1], Camila Laís Gonçalves Ribeiro[1]; Jacqueline Gonçalves dos Santos[1]; Anderson Fraga da Cruz[1], João Luiz Aldinucci Buzzo[1], Helyn Barddal[2], Thales R. Cipriani[2], Marcelo Dias-Baruffi[3], Edvaldo da Silva Trindade[1], Fernanda Fogagnoli Simas[1], Carolina Camargo de Oliveira[1]**

[1] Cell Biology department, Universidade Federal do Paraná (UFPR), Curitiba, 81531-980, Brazil.

[2] Biochemistry department, Universidade Federal do Paraná (UFPR), Curitiba, 81531-980, Brazil.

[3] School of Pharmaceutical Sciences of Ribeirão Preto, Universidade de São Paulo (USP), Ribeirão Preto, 14040-903, Brazil

‡ These authors contributed equally

* Corresponding authors – e-mail addresses: ferfs@ufpr.br (F.F. Simas); krokoli@ufpr.br (C. Camargo de Oliveira)

Supplementary Table 1. *In Vivo* Organ Weight of GGHS and GGH.

Group	Organ Weight (g)					
	Kidneys	Liver	Heart	Spleen	Lung	Tumor
Control (PBS)	1.39 ± 0.06	5.35 ± 0.30	0.54 ± 0.08	0.36 ± 0.04	0.81 ± 0.11	1.86 ± 1.61
GGH 5 mg/kg	1.43 ± 0.03	5.35 ± 0.34	0.52 ± 0.05	0.40 ± 0.05	0.84 ± 0.11	1.32 ± 0.91
GGHS 5 mg/kg	1.43 ± 0.10	5.35 ± 0.10	0.52 ± 0.02	0.39 ± 0.05	0.78 ± 0.11	2.16 ± 1.49

Data are presented as mean ± standard deviation. The Kruskal-Wallis test followed by Dunn's multiple comparisons test was employed ($p<0.05$).

Supplementary Table 2. Blood Test - Biochemical Parameters Analysis.

Group	Biochemical Blood			
	mg/dL		U/L	
	ALT/GPT	AST/GOT	Creatinine	Urea
Control (PBS)	51.00 ± 3.94	556.00 ± 508	0.26 ± 0.08	62.70 ± 30.40
GGH 5 mg/kg	45.30 ± 5.43	342.00 ± 85.9	0.22 ± 0.07	35.70 ± 0.76
GGHS 5 mg/kg	55.30 ± 5.15	665.00 ± 444	0.25 ± 0.05	48.10 ± 10.70

ALT – Alanine Aminotransferase Enzyme

GPT – Glutamic-Pyruvic Transaminase Enzyme

AST – Aspartate Aminotransferase Enzyme

GOT – Oxalacetic Transaminase Enzyme

Data are presented as mean ± standard deviation. The Kruskal-Wallis test followed by Dunn's multiple comparisons test was employed ($p<0.05$).

Supplementary Table 3. Blood Test - Erythrocyte Series Analysis.

Group	Complete Blood Count - Erythrocyte Series						
	pg		%		fL	g/dL	10^6 / uL
	MCH	MCHC	HCT	RDW	MCV	HBG	RBC
Control (PBS)	13.60 ± 0.19	33.00 ± 0.23	30.70 ± 4.75	12.90 ± 1.33	41.30 ± 0.78	9.61 ± 2.08	7.44 ± 1.23
GGH 5 mg/kg	13.50 ± 0.27	32.70 ± 0.85	29.40 ± 4.43	13.30 ± 1.20	41.50 ± 1.10	9.61 ± 1.51	7.11 ± 1.19
GGHS 5 mg/kg	13.60 ± 0.30	32.70 ± 0.25	27.70 ± 4.69	13.50 ± 1.50	41.50 ± 0.90	9.03 ± 1.58	6.64 ± 1.34

MCH – Mean Corpuscular Hemoglobin

MCHC – Mean Corpuscular Hemoglobin Concentration

HCT – Hematocrit

RDW – Red Blood Cell Distribution Width

MCV – Mean Corpuscular Volume

HBG – Hemoglobin

RBC – Total Red Blood Cell Count

Data are presented as mean ± standard deviation. The Kruskal-Wallis test followed by Dunn's multiple comparisons test was employed ($p<0.05$).

Supplementary Table 4. Blood Test - Leukocyte Series Analysis.

Group	Complete Blood Count - Leukocyte Series						
	%			10^3 / uL			
	LINF	MON	GRAN	WBC	LINF	MON	GRAN
Control (PBS)	69.60 ± 10.10	2.65 ± 0.84	27.80 ± 9.46	1.88 ± 0.90	1.29 ± 0.57	0.05 ± 0.05	0.54 ± 0.37
GGH 5 mg/kg	68.40 ± 10.10	2.70 ± 0.54	28.90 ± 6.73	1.65 ± 0.68	1.15 ± 0.56	0.02 ± 0.05	0.47 ± 0.19
GGHS 5 mg/kg	68.90 ± 7.66	3.23 ± 1.05	27.90 ± 6.68	2.35 ± 1.61	1.64 ± 1.20	0.06 ± 0.05	0.65 ± 0.41

LINF – Lymphocytic Cells

MON – Mononuclear Cells

GRAN – Granulocytic Cells

WBC – White Blood Cells

Data are presented as mean ± standard deviation. The Kruskal-Wallis test followed by Dunn's multiple comparisons test was employed ($p<0.05$).

Supplementary Table 5. Blood Test - Platelet Series Analysis.

Grupo	Complete Blood Count - Platelet Series			
	10^3/uL	fL	-	%
	PLT	MPV	PDW	PCT
Control (PBS)	414.00 ± 49.00	7.71 ± 2.28	15.70 ± 0.17	0.28 ± 0.03
GGH 5 mg/kg	379.00 ± 92.60	6.88 ± 0.66	15.50 ± 0.21	0.26 ± 0.06
GGHS 5 mg/kg	382.00 ± 43.10	6.51 ± 0.39	15.40 ± 0.30	0.25 ± 0.03

PLT – Platelet Count

MPV – Mean Platelet Volume

PDW – Platelet Distribution Width

PCT – Plateletcrit

Data are presented as mean ± standard deviation. The Kruskal-Wallis test followed by Dunn's multiple comparisons test was employed ($p<0.05$).

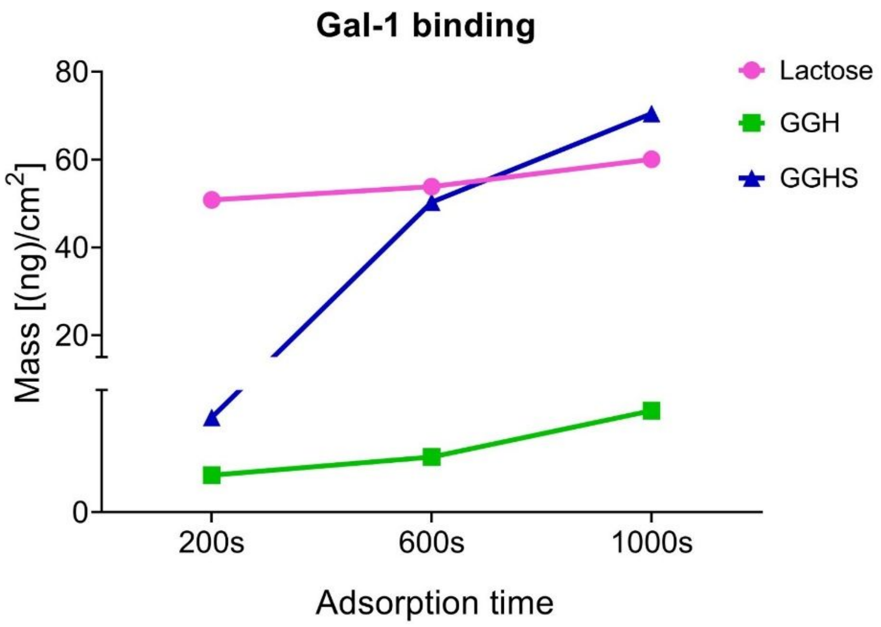

FIGURE S1: **Galectin-1 binding assay by QCM-D (quartz crystal microbalance with dissipation monitoring) analysis.** GGH (100 µg/mL) and GGHS (1000 µg/mL) were subjected to binding assay to galectin-1 that was bound to the balance sensor. Lactose (10 mg/mL) was used as a positive binding control. Graph displaying three measured adsorption timepoints (200s, 600s, and 1000s).

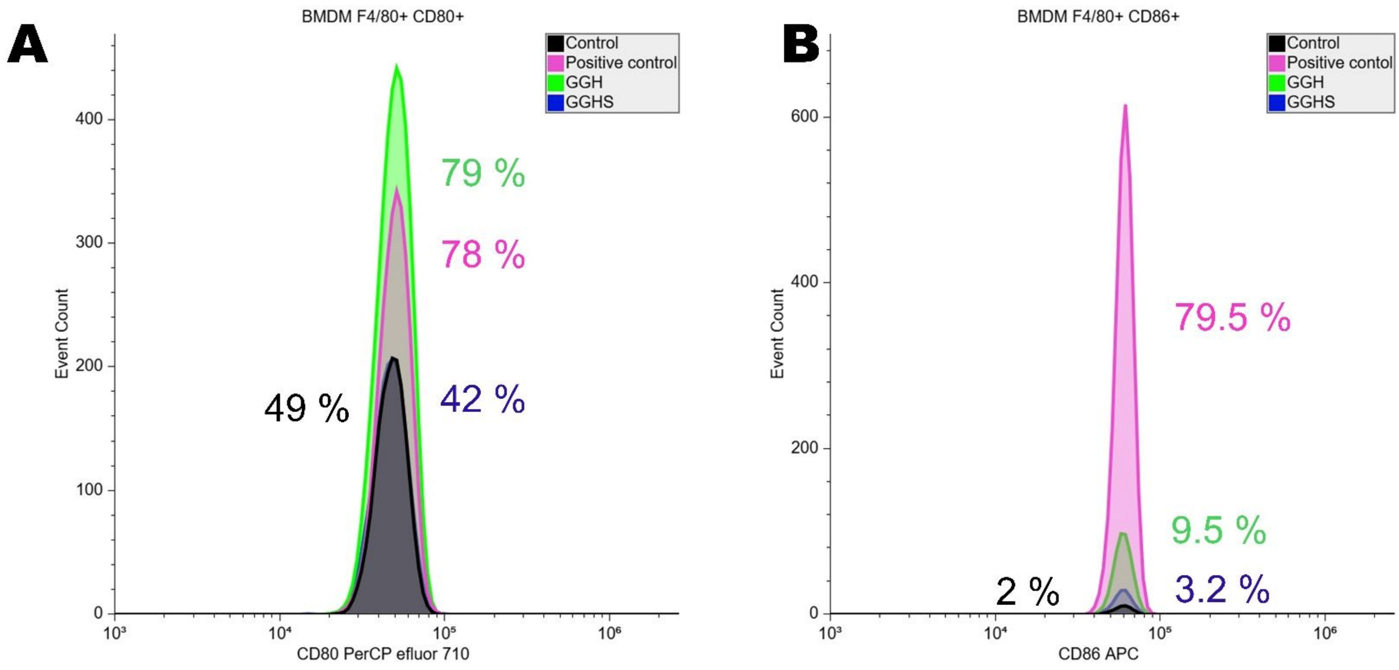

FIGURE S2: BMDM M1 polarization by GGH. Overlay histogram of data of F4/80+ cells (macrophages) and CD80+ cells **(A)** and CD86+ cells **(B)**. Percentage of positive control (pink) for CD80+ and CD86+ marker in 20 ng/mL IFNγ + 100 ng/m LPS induced BMDM cells, control group (black), GGH (green), and GGHS (blue), are shown for each condition **(A)** CD80+ and **(B)** CD86+. Data represents a sample from a pool of bone marrow cells obtained from five animals.
